# Bovine Endometrial Epithelial Cells Scale Their Pro-inflammatory Response *In vitro* to Pathogenic *Trueperella pyogenes* Isolated from the Bovine Uterus in a Strain-Specific Manner

**DOI:** 10.3389/fcimb.2017.00264

**Published:** 2017-06-21

**Authors:** Mohammad Ibrahim, Sarah Peter, Karen Wagener, Marc Drillich, Monika Ehling-Schulz, Ralf Einspanier, Christoph Gabler

**Affiliations:** ^1^Institute of Veterinary Biochemistry, Department of Veterinary Medicine, Freie Universität BerlinBerlin, Germany; ^2^Clinical Unit for Herd Health Management in Ruminants, University Clinic for Ruminants, Department for Farm Animals and Veterinary Public Health, University of Veterinary Medicine ViennaVienna, Austria; ^3^Functional Microbiology, Institute of Microbiology, Department for Pathobiology, University of Veterinary Medicine ViennaVienna, Austria

**Keywords:** *Trueperella pyogenes*, endometritis, bovine endometrial cells, immune cells, FTIR spectroscopy

## Abstract

Among different bacteria colonizing the bovine uterus, *Trueperella pyogenes* is found to be associated with clinical endometritis (CE). The ability of cows to defend against *T. pyogenes* infections depends on the virulence of invading bacteria and on the host's innate immunity. Therefore, to gain insights into bacterial factors contributing to the interplay of this host pathogen, two strains of *T. pyogenes* were included in this study: one strain (TP2) was isolated from the uterus of a postpartum dairy cow developing CE and a second strain (TP5) was isolated from a uterus of a healthy cow. The two strains were compared in terms of their metabolic fingerprints, growth rate, virulence gene transcription, and effect on bovine endometrial epithelial cells *in vitro*. In addition, the effect of the presence of peripheral blood mononuclear cells (PBMCs) on the response of endometrial epithelial cells was evaluated. TP2, the strain isolated from the diseased cow, showed a higher growth rate, expressed more virulence factors (*cbpA, nanH, fimE*, and *fimG*), and elicited a higher mRNA expression of pro-inflammatory factors (*PTGS2, CXCL3*, and *IL8*) in bovine endometrial epithelial cells compared with TP5, the strain isolated from the healthy cow. The presence of PBMCs amplified the mRNA expression of pro-inflammatory factors (*PTGS2, CXCL3, IL1A, IL6*, and *IL8*) in bovine endometrial epithelial cells co-cultured with live TP2 compared with untreated cells, especially as early as after 4 h. In conclusion, particular strain characteristics of *T. pyogenes* were found to be important for the development of CE. Furthermore, immune cells attracted to the site of infection might also play an important role in up-regulation of the pro-inflammatory response in the bovine uterus and thus significantly contribute to the host-pathogen interaction.

## Introduction

The subfertility of high-producing dairy cows represents a major obstacle to the profitability and sustainability of the dairy industry. Among the most common reasons for subfertility are postpartum uterine diseases caused by uterine bacterial infections after parturition (Sheldon et al., 2009). During the postpartum period, up to 50% of all dairy cows develop uterine inflammatory diseases, such as metritis, and clinical or subclinical endometritis (Sheldon et al., 2009). The incidence of uterine inflammation is associated with the presence of certain bacterial species in the uterus, such as *Escherichia coli, Trueperella pyogenes, Fusobacterium* spp., and *Bacteroides* spp. (Williams et al., 2005; Sheldon et al., 2010; Machado et al., 2012; Wagener et al., 2015). In particular, *T. pyogenes* has been found to be associated with chronic and severe forms of endometritis (Bonnett et al., 1991; Wagener et al., 2014b).

*T. pyogenes* is a gram-positive opportunistic bacterium that acts as a primary uterine pathogen (Amos et al., 2014; Lima et al., 2015). The pathogenicity of *T. pyogenes* is attributed to pyolysin (PLO), which causes cytolysis of host cells (Jost et al., 1999; Amos et al., 2014). Moreover, *T. pyogenes* expresses a number of known and putative virulence factors, which may be involved in adhesion to host cells (Jost and Billington, 2005). Furthermore, *T. pyogenes* could trigger a pro-inflammatory response within the uterus, with transmigration of neutrophils and evidence of mucopurulent discharge (Amos et al., 2014; Lima et al., 2015). The establishment of uterine bacterial infections depends on the pathogenic potential of invading bacteria and the local uterine immune response. Endometrial epithelial cells, as the first line of defense, can initiate an immune reaction by increased synthesis of different cytokines [interleukin 1A (*IL1A*), *IL6, IL8*, and CXC ligand 3 (*CXCL3*)] and prostaglandin endoperoxide synthase 2 (*PTGS2*; Amos et al., 2014; Turner et al., 2014; Gärtner et al., 2016), leading to an influx of immune cells to the site of infection (Takeuchi and Akira, 2010). Although *T. pyogenes* is associated with severe endometritis, it has also been isolated from cows without signs of uterine disease at puerperium (Silva et al., 2008; Santos et al., 2010; Wagener et al., 2015).

The effect of uterine pathogens on endometrial epithelial cells has been studied extensively using *in vitro* models (Davies et al., 2008; MacKintosh et al., 2013; Gärtner et al., 2016). These models have used epithelial cells and/or stromal cells. However, this may not reflect the complex host-pathogen interactions *in vivo*, which involve further interactions between endometrial epithelial cells and immune cells. In addition, endometrial explants have been used, but in this model it is not possible to discriminate between the distinct influences of each cell type (Borges et al., 2012).

Therefore, the aim of this study was to reveal the mechanisms of host-pathogen interactions in the bovine endometrium *in vitro* that may be associated with the establishment of uterine diseases. The objectives were to characterize a *T. pyogenes* strain isolated from the uterus of a cow developing clinical endometritis (CE) and a strain from a healthy cow to assess the importance of strain-specific factors for the development of bovine CE. Further objectives were to examine the viability and mRNA expression of pro-inflammatory factors of bovine endometrial epithelial cells in the presence of (1) two different strains of *T. pyogenes*, including live bacteria, and bacterial endo- and exo-toxins, and (2) peripheral blood mononuclear cells (PBMCs) in an *in vitro* model extending the epithelial cell co-culture system with *T. pyogenes*. PBMCs were chosen to mimic some aspects of a chronic uterine inflammation caused by *T. pyogenes* infection that involves the infiltration of lymphocytes. In addition, PBMCs were evaluated for their response in the presence of *T. pyogenes* concerning the viability and mRNA expression of selected pro-inflammatory factors.

## Materials and methods

All animal experimental procedures were carried out in accordance with the European Community Directive 86/609/EEC and were approved by the Ethics Committee of the University of Veterinary Medicine Vienna (08/01/97/2011; date of approval February 1, 2011) and the responsible state veterinarian in Schleswig-Flensburg (Schleswig-Holstein, Germany). As only abattoir waste was used for the cell culture experiments, there was no need to adhere to institutional or national research council guidelines.

### Cultivation and preparation of bacteria

Intrauterine bacteriological samples were collected using the cytobrush technique from Holstein-Friesian cows on a commercial dairy farm in Schleswig-Holstein (Germany), as described previously (Wagener et al., 2014b, 2015). The strains of *T. pyogenes* used in this study were isolated from cows on day 15 postpartum (pp). One *T. pyogenes* strain (TP2) was isolated from a cow that showed vaginal discharge with more than 50% pus on day 21 pp and was classified accordingly as a cow developing CE. Another *T. pyogenes* strain (TP5) was isolated from a cow that showed clear vaginal discharge on day 21 pp and was classified accordingly as a healthy cow. The bacteria were cultivated in 5 ml brain heart infusion (BHI) broth (Fluka, Steinheim, Germany), supplemented with 5% heat-inactivated (HI) fetal calf serum (FCS; Biochrom, Berlin, Germany) at 37°C for 48 h under aerobic conditions. The bacteria were harvested by centrifugation at 2,000 × g for 10 min and washed once with Dulbecco's phosphate-buffered saline (PBS; Sigma-Aldrich, Deisenhofen, Germany). Both strains (TP2, TP5) were stored in aliquots containing 80% (v/v) bacteria suspended in PBS and 20% (v/v) glycerol at −80°C until further use. Some aliquots were thawed and plate counting was performed on sheep blood agar (Oxoid, Hampshire, United Kingdom) to determine the number of colony-forming units (CFU)/ml.

Heat-inactivated (HI) bacteria and bacteria-free filtrates (BFF) of both *T. pyogenes* strains (TP2, TP5) were prepared by enriching them from a glycerol stock in BHI supplemented with 5% FCS for 48 h at 37°C. Bacteria were harvested by centrifugation at 2,000 × g, washed with PBS and incubated in epithelial cell culture medium [Dulbecco's Modified Eagle Medium (DMEM)/Ham's F-12 medium containing 10% FCS (both Biochrom)] at 37°C and 5% CO_2_ until the medium changed color to yellow. To heat inactivate the bacteria, a portion of this bacterial suspension was incubated at 63°C for 30 min, centrifuged at 2,000 × g for 10 min, washed once with PBS, re-suspended in PBS, and stored in aliquots at −80°C until further use. To obtain the BFF, another portion of the bacterial suspension was centrifuged at 2,000 × g for 10 min. The supernatant was filtered through a 0.22 μm filter (Rotilabo syringe filters, Carl Roth, Karlsruhe, Germany) and stored in aliquots at −80°C until further use. Aliquots of the bacterial suspension were used for plate counting on sheep blood agar (Oxoid) to determine the number of CFU/ml for HI bacteria or equivalents to BFF. A sample of the prepared HI bacteria and BFF was cultured on sheep blood agar plates and incubated at 37°C for 48 h. Absence of bacterial colonies indicated complete inactivation and absence of any live bacteria.

### Bacterial growth measurements

Both *T. pyogenes* strains (TP2 and TP5), reactivated from glycerol stocks, were pre-grown in 5 ml BHI broth supplemented with 5% HI FCS at 37°C under aerobic conditions for 48 h. The optical density of each bacterial suspension was measured at the wavelength of 600 nm (OD_600_) and bacteria were diluted into 50 ml BHI broth supplemented with 5% HI FCS to obtain a starting OD_600_ of 0.05, which was confirmed by OD_600_ measurement. Bacterial cultures were incubated at 37°C under aerobic conditions and OD_600_ was measured every 2 h over a period of 24 h. The experiment was repeated independently five times.

In addition, samples from three of the independent growth experiments were taken after 6, 12, and 24 h for transcriptional analyses of selected virulence genes, as described in detail below. Cells were harvested by centrifugation at 2,000 × g for 10 min and washed once with PBS. The resulting bacterial cell pellets were stored at −80°C until further use.

### Fourier transform infrared (FTIR) spectroscopy analysis

FTIR spectroscopy was employed to generate bacterial metabolic fingerprints, as described previously (Ehling-Schulz et al., 2005). Briefly, bacteria were grown as lawns on tryptic soy agar (Oxoid) for 24 h at 30°C. FTIR measurements were carried out using a Tensor 27 FTIR spectrometer (Bruker Optics, Billerica, MA, USA) coupled to a HTS-XT microplate adapter. Data analysis of FTIR spectra was performed using the OPUS software (version 5.5; Bruker Optics). First derivatives of the original spectra were calculated and spectral windows of 3,030 to 2,830, 1,350 to 1,200, and 900 to 700 cm^−1^ were used with a weight factor of 1 and a repro-level 30 for the cluster analysis. Hierarchical cluster analysis (HCA) was performed as described previously (Wagener et al., 2014a). Dendrograms were calculated using Ward's algorithm and *Streptococcus uberis* as an outgroup. To identify the spectral regions with the most significant differences between TP2 and TP5, normalized average spectra of the three independent FTIR measurements were calculated for both strains, and the average spectra of TP2 and TP5 were compared.

### Screening for the presence of selected virulence genes in the genome of different *T. pyogenes* strains

Bacterial genomic DNA was extracted using the RTP Bacteria DNA Mini Kit (Stratec, Berlin, Germany) according to the manufacturer's instructions. For this purpose, cell pellets obtained from the growth curve experiments of the two different *T. pyogenes* strains were used. DNA yield was estimated by spectrophotometry at a wavelength of 260 nm.

A conventional PCR was carried out, as described previously (Gärtner et al., 2016), using 150 ng of genomic bacterial DNA. Primer pairs were designed using Primer-BLAST (Ye et al., 2012) and synthesized with Eurofins Genomics (Ebersberg, Germany). The amplification was performed using cycling conditions as follows: 10 min at 95°C, 40 cycles of 15 s at 95°C, 30 s at the specific annealing temperature for each primer pair (Table 1) and 30 s at 72°C, followed by a final extension step at 72°C for 4 min. PCR without DNA served as a negative control. The identity of each amplicon was further confirmed by DNA sequencing (GATC Biotech, Konstanz, Germany). Based on sequence data, specific primers for *fimA, fimC, fimE*, and *fimG* (Table 1) were designed for the subsequent quantitative PCR.

**Table 1:** Selected known and putative *T. pyogenes* virulence gene transcripts, primer sequences, and annealing temperatures used for PCR/qPCR with expected amplicon size.

**Gene symbol**	**Accession no./references**	**Primer sequence (5′ → 3′)**	**Annealing temperature**	**Amplicon size (bp)**
*plo*	AB027461.1	F: AAG TAT CCT GAC CAT GCT GC	60°C	228
		R: GCC GAA AAC GCT ATG TGG AG		
*cbpA*	AY223543.1	F: TAC TGT TCG TCC AAC TCG CAT C	60°C	110
		R: TGC CCG GCT TGA TAT AAC CTT C		
*nanH*	AF298154.1	F: CAC GGA CGT GAA GAG CTT TG	63°C	243
		R: ACT TCA ACC TTC GGC TCT GG		
*nanP*	AY045771.1	F: GAT GAC GCA AAC AAG ACG CC	60°C	152
		R: GTC AGC ACA AAA CCA GCC AG		
*fimA*	Silva et al., 2008	F: CAC TAC GCT CAC CAT TCA CAA G	57°C	605
		R: GCT GTA ATC CGC TTT GTC TGT G		
*fimA-Q*	this study	F: CCG TTC CTC GTT ACC CTT CC	60°C	152
		R: CAG GTA ATC TCA GCA CCG GG		
*fimC*	Silva et al., 2008	F: TGT CGA AGG TGA CGT TCT TCG	60°C	843
		R: CAA GGT CAC CGA GAC TGC TGG		
*fimC-Q*	this study	F: GCC GTT CGC TTC ACA CTT AC	60°C	137
		R: ATG GCA AAA CCA AAG ACG CC		
*fimE*	Silva et al., 2008	F: GCC CAG GAC CGA GAG CGA GGG C	55°C	775
		R: GCC TTC ACA AAT AAC AGC AAC C		
*fimE-Q*	this study	F: CGC CCG TTC TTC TTT GCT TC	60°C	143
		R: TGC CTC GTT GAG ACC AAG TC		
*fimG*	Silva et al., 2008	F: ACG CTT CAG AAG GTC ACC AGG	57°C	929
		R: ATC TTG ATC TGC CCC CAT GCG		
*fimG-Q*	this study	F: GTA GCC GGA GTT GAG GAA GG	60°C	157
		R: ATC CTC GCT CTC TTG CTG TG		
*16S rRNA*	NR_044858.1	F: AAG ACC GGG GCT TAA CTT CG	60°C	133
		R: AGT AAC CTG CCT TCG CCA TC		
*smc*	U84782.2	F: ATG ATC ACA CTC CCG CAA CC	60°C	119
		R: GGG TTG ATC TTG CCC AAA CG		

### Primary bovine endometrial epithelial cell culture

Bovine endometrial epithelial cells were isolated and cultured as described previously (Betts and Hansen, 1992; Gärtner et al., 2015). Briefly, apparent healthy uteri from non-pregnant cows were obtained from a local abattoir. Small pieces of the endometrium were cut from various loci in the intercaruncular regions. The tissue mass was chopped very finely before being digested in an enzyme solution composed of 150 U/ml collagenase (Sigma-Aldrich), 150 U/ml hyaluronidase (Sigma-Aldrich), 200 U/ml penicillin (Biochrom), and 200 μg/ml streptomycin (Biochrom) in Hank's Balanced Salt Solution (Biochrom) for 2 h at 37°C. After centrifugation, the cell pellet obtained was washed in epithelial cell culture medium (DMEM/Ham's F-12 medium containing 10% FCS, 55 μg/ml gentamicin, and 1.4 μg/ml amphotericin B; all from Biochrom). Then, the cells were seeded in 25 cm^2^ flasks (Corning, Corning, NY, USA) and incubated in a humidified incubator at 37°C and 5% CO_2_ for 18 h to allow selective attachment of stromal cells. After this time, the medium containing unattached cells was re-seeded to obtain a pure epithelial cell population (Sheldon et al., 2010).

In the first passage, endometrial epithelial cells were cultured until they reached more than 80% confluence. At that time, they were seeded into 24-well plates at a density of 1 × 10^5^ cells in 0.5 ml culture medium or in 6-well plates at a density of 3 × 10^5^ cells in 3 ml culture medium.

After the endometrial epithelial cells reached confluence in the second passage, they were prepared for co-culture experiments by removing the medium and washing twice with PBS.

Immunocytochemistry against pan-keratins was performed as described previously (Miessen et al., 2012). After the second passage, a 100% pure epithelial cell population was observed.

### PBMC isolation and culturing

Whole cow blood collected at the slaughterhouse was used for PBMC isolation by density gradient centrifugation using Ficoll-Paque plus (GE Healthcare, Uppsala, Sweden). The isolated PBMCs were pre-incubated with epithelial cell culture medium at a density of 5 × 10^6^ cells/ml for 24 h in a humidified incubator at 37°C and 5% CO_2_ to adjust the cells to culture conditions.

After the 24 h incubation, the PBMCs were washed twice with PBS and suspended in antibiotic-free epithelial cell culture medium before co-culturing with endometrial epithelial cells and/or *T. pyogenes*.

### Co-culture experiments

In experiment 1, endometrial epithelial cells (*n* = 5 cows) were co-cultured in passage 2 in 24-well plates with bacterial strain TP2 or TP5 in the form of live bacteria at a multiplicity of infection of 1 (MOI = 1), HI bacteria at MOI = 1, or BFF equivalent to MOI = 1.

In experiment 2, endometrial epithelial cells were co-cultured (*n* = 5 cows) in 6-well plates with live TP2 at MOI = 1 and/or heterogeneous PBMCs at a ratio of 1:1 to endometrial epithelial cells.

Different treatments were applied using antibiotic-free epithelial cell culture medium. Epithelial cells cultured with epithelial cell culture medium without antibiotics served as controls. After the indicated time points in **Figures 5**, **6**, the medium was removed, and the epithelial cells were washed twice with PBS and lysed with RLT lysis buffer (Qiagen, Hilden, Germany). Control epithelial cells were also lysed at 0 h. Cell lysates were stored at −80°C until further use. In addition, the viability of the epithelial cells (*n* = 3) was monitored in 24-well plates for all treatments and corresponding controls by Trypan blue staining up to 72 h of co-culture, as described previously (Gärtner et al., 2016). Different optical fields were considered, pictures were taken and analyzed using ImageJ version 1.51 (National Institutes of Health, USA). The number of viable (unstained) cells was calculated as the percentage of the total number of cells.

In experiment 3, the hypothesis that PBMCs can influence the growth of *T. pyogenes* in lower concentrations was tested. PBMCs (*n* = 3 cows) were seeded after pre-incubation in 6-well plates at a density of 1 × 10^7^ cells in antibiotic-free epithelial cell culture medium and co-cultured with TP2 at MOI = 0.1. After 2, 4, and 6 h, PBMCs were harvested by centrifugation at 850 × g for 5 min and washed once with PBS. The cell pellets were stored at −80°C until use. In addition, the viability of the PBMCs (*n* = 3) co-cultured with TP2 at two different MOI (0.1 and 1) was monitored up to 48 h using a Neubauer chamber and Trypan blue exclusion.

### Total RNA extraction and reverse transcription

Total RNA was isolated from lysed co-cultured endometrial epithelial cells, as described previously (Gärtner et al., 2016). Lysed PBMCs were subjected to total RNA extraction using the InviTrap Spin Cell RNA Mini Kit (Stratec) according to the manufacturer's instructions.

Bacterial total RNA was extracted using the RNeasy Plus Mini Kit (Qiagen) according to the manufacturer's protocol with some modifications. Briefly, a frozen bacterial cell pellet was suspended in 100 μl 10 mM Tris-HCl—1 mM EDTA buffer (pH 8). Then, 500 μl lysis buffer RLT (Qiagen) containing 1% of 2-mercaptoethanol (Carl Roth) was added to the cell suspension and this was transferred in its entirety to a 2-ml screw cap microtube (Sarstedt, Nümbrecht, Germany) containing 300 mg of 0.1 mm silica spheres (Lysing Matrix B; MP Biomedicals, Eschwege, Germany). The bacterial cells were lysed by shaking the tube using a Fastprep machine (FP120, Savant Instruments, New York, USA) at maximum speed for 30 s for 4 cycles with 2 min cooling on ice between consecutive cycles. The cell debris was removed by centrifugation at 11,000 × g for 1 min and the supernatant containing total RNA was loaded on an RNeasy spin column.

The total RNA yield from eukaryotic and bacterial cells was estimated spectrophotometrically at 260 nm. RNA integrity and quality was verified using an RNA 6000 Nano LabChip kit on an Agilent 2100 Bioanalyzer (both Agilent Technologies, Waldbronn, Germany).

Reverse transcription (RT) was performed as described previously (Odau et al., 2006). Briefly, 150 ng total RNA from eukaryotic or bacterial cells was treated with DNAse I (Fermentas, St. Leon-Roth, Germany) in a first step to remove any genomic DNA contamination (Huang et al., 1996). In the second step, first-strand cDNA was synthesized from total RNA using 2.5 μM random hexamers, 0.66 mM dNTPs, 1 × RT buffer and 200 U RevertAid reverse transcriptase (all from Fermentas) in a total volume of 60 μl. Each sample was stored as 20 μl aliquots at −20°C until further use. Reactions without reverse transcriptase were run in parallel to the RT to confirm the absence of any genomic DNA or contamination.

### Quantitative PCR (qPCR)

qPCR was carried out as described previously (Odau et al., 2006) following the minimum information for publication of quantitative real-time PCR experiments (MIQE) guidelines (Bustin et al., 2009). Briefly, 1 μl cDNA was amplified in the presence of 1x SensiMix Low-ROX (Bioline, Luckenwalde, Germany) and 0.4 μM of each primer (Tables 1, 2) in a total volume of 10 μl using the Rotor Gene 3000 (Corbett Research, Mortlake, Australia). The qPCR was performed with the following temperature profile: an initial denaturation step at 95°C for 10 min, followed by a cycling step (45 cycles, each cycle consisting of 15 s denaturation at 95°C, 20 s annealing at temperature specified for each gene (Tables 1, 2), and 30 s extension at 72°C), followed by a melting curve program (temperature ramp starting from 50 to 99°C with continuous fluorescence monitoring), and a final cooling step at 40°C for 1 min.

**Table 2:** Selected bovine gene transcripts, primer sequences, and annealing temperatures used for quantitative PCR with expected amplicon size.

**Gene symbol**	**Accession no./references**	**Primer sequence (5′ → 3′)**	**Annealing temperature**	**Amplicon size (bp)**
*18S rRNA*	Odau et al., 2006	F: GAG AAA CGG CTA CCA CAT CCA A	61°C	337
		R: GAC ACT CAG CTA AGA GCA TCG A		
*ACTB*	Gärtner et al., 2015	F: CGG TGC CCA TCT ATG AGG	58°C	266
		R: GAT GGT GAT GAC CTG CCC		
*GAPDH*	Gärtner et al., 2015	F: CCC AGA AGA CTG TGG ATG G	62°C	306
		R: AGT CGC AGG AGA CAA CCT G		
*SDHA*	Gärtner et al., 2015	F: GGG AGG ACT TCA AGG AGA GG	60°C	219
		R: CTC CTC AGT AGG AGC GGA TG		
*CXCL3*	Gärtner et al., 2016	F: GCC ATT GCC TGC AAA CTT	56°C	189
		R: TGC TGC CCT TGT TTA GCA		
*IL8*	Fischer et al., 2010	F: CGA TGC CAA TGC ATA AAA AC	56°C	153
		R: CTT TTC CTT GGG GTT TAG GC		
*IL1A*	Gabler et al., 2009	F: TCA TCC ACC AGG AAT GCA TC	59°C	300
		R: AGC CAT GCT TTT CCC AGA AG		
*IL6*	Konnai et al., 2003	F: TCC AGA ACG AGT ATG AGG	56°C	236
		R: CAT CCG AAT AGC TCT CAG		
*PTGS2*	Odau et al., 2006	F: CTC TTC CTC CTG TGC CTG AT	60°C	359
		R: CTG AGT ATC TTT GAC TGT GGG AG		

To quantify the content of each expressed gene of interest, a serial dilution of PCR products with a known concentration of the target gene was amplified simultaneously with the samples, generating a standard curve. In relation to the standard curve generated, concentrations of target genes were calculated using the Rotor Gene 6.1 software (Corbett Research). The specificity of amplification of target genes was confirmed by melting point analysis using the Rotor Gene 6.1 software (Corbett Research) and by sequencing (GATC Biotech) of the obtained amplicons, which showed a 100% homology to the published sequences or to the bacterial sequences already obtained in the study.

### Statistical analysis

To normalize the qPCR data obtained, the mRNA expression values of pro-inflammatory factors generated in mammalian cells were divided by an accurate normalization factor. This factor was calculated using geNorm (Vandesompele et al., 2002) for the most stable expressed reference genes in the endometrial epithelial cells [18S ribosomal RNA (*18S rRNA*), glyceraldehyde 3-phosphate dehydrogenase (*GAPDH*), and succinate dehydrogenase complex, subunit A (*SDHA*)], in PBMCs [*18S rRNA*, beta actin (*ACTB*), and *SDHA*], and in bacteria [*16S rRNA* and chromosome segregation protein (*smc*)].

Due to the large number of samples of endometrial epithelial cells co-cultured with different strains of *T. pyogenes*, the target genes were amplified in more than one run. Therefore, the normalized values obtained from these cells were inter-run calibrated by dividing using an inter-calibration factor, which was calculated by the geometric averaging of the normalized mRNA expression values of 10 inter-run calibrator samples (Hellemans et al., 2007).

The normalized values obtained for pro-inflammatory factor mRNA expression in the endometrial epithelial cells co-cultured with *T. pyogenes* and/or PBMCs, and PBMCs and the normalized mRNA expression values of virulence genes in different *T. pyogenes* strains were log-transformed.

Normalized inter-run calibrated values and normalized log-transformed values in controls and treatments at each time point were scaled relative to the mRNA expression value at 0 h, set equal to one. These relative values were used to generate bar charts, presenting means ± SEM.

An independent *t*-test was undertaken to compare the differences between the growth rate and normalized mRNA expression of the virulence genes of two *T. pyogenes* strains (TP2 vs. TP5) at different time points. One-way repeated measures analysis of variance (ANOVA) with the Bonferroni *post-hoc* test was used to compare the differences between the normalized mRNA expression of virulence factors at three different time points within each *T. pyogenes* strain. The Wilcoxon signed-rank test was used to calculate statistical differences between the treatments with different *T. pyogenes* strains. For this, each treatment was compared with the control at the same time point, and the same treatments of each strain (TP2 vs. TP5) were also compared. General linear model multivariate ANOVA with the Bonferroni *post-hoc* test was used for multiple comparisons of different treatments with *T. pyogenes* and/or PBMCs. A paired *t*-test was performed to compare the effect of *T. pyogenes* on PBMCs (untreated vs. treated).

All statistical calculations were undertaken using SPSS version 22 (SPSS, Chicago, USA). Values of *P* ≤ 0.05 were considered to be significant; *P*-values of 0.05–0.1 were considered to indicate tendencies.

## Results

### Growth of the two different *T. pyogenes* strains

**Figure 1:**
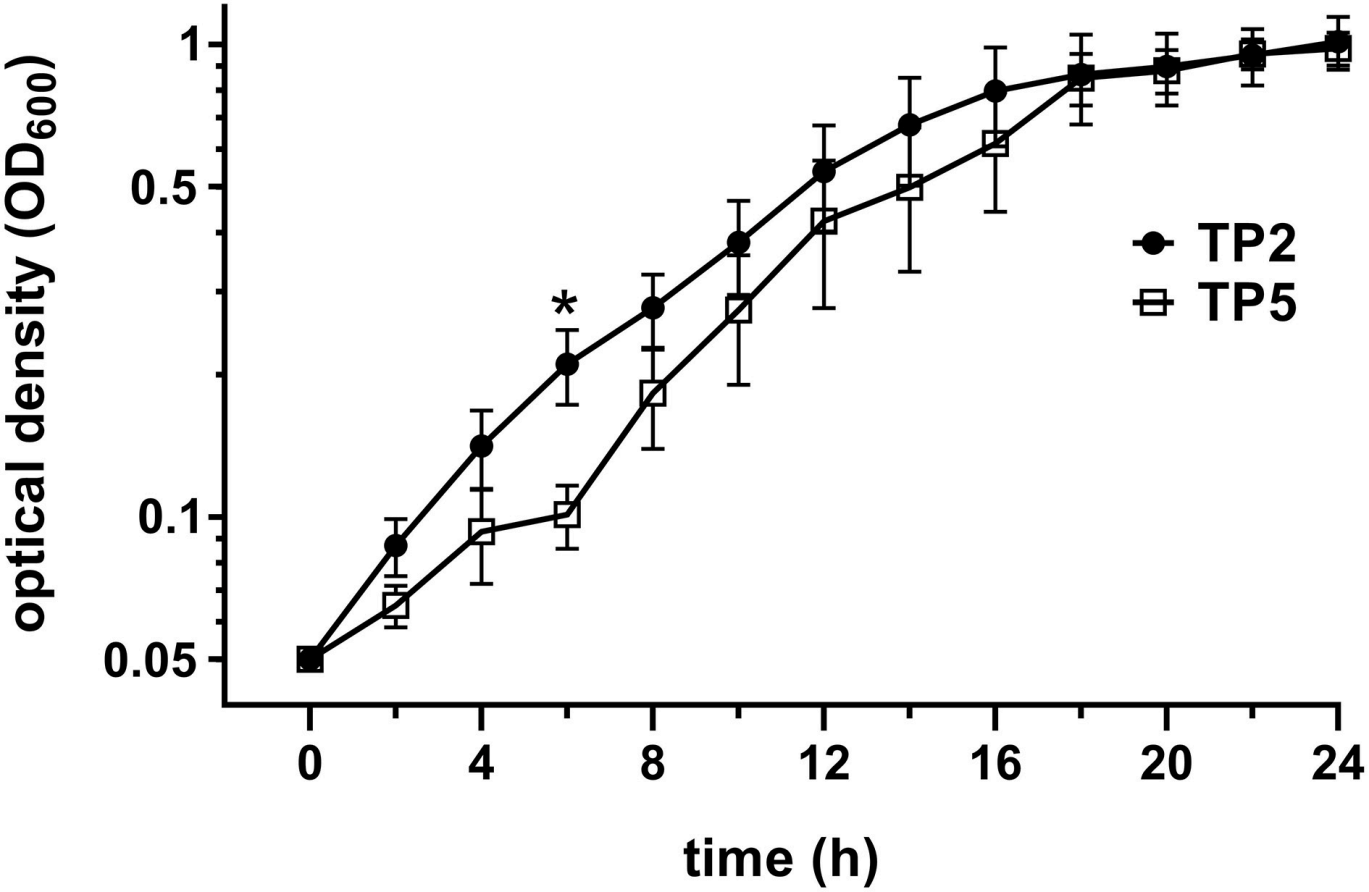
Comparison of standard growth curves of TP2 and TP5. Standard growth curves were generated by measuring OD_600_ by spectrophotometer every 2 h for 24 h. Data are presented as mean ± SEM of OD_600_ readings for 5 different inocula for each strain. Asterisk indicates a significant difference (*P* < 0.05) between the two strains at that time point.

*T. pyogenes* strain TP2 grew faster than TP5 during the first hours (Figure 1), reaching a significant difference at 6 h (*P* = 0.043). However, both strains reached their plateau phase of growth after 18 h. In addition, TP2 grew as a homogeneous suspension during the entire 24 h period in contrast to TP5, which grew up to 14 h rather as a cloudy suspension and thereafter as a homogeneous one.

### Metabolic fingerprints of TP2 and TP5

**Figure 2:**
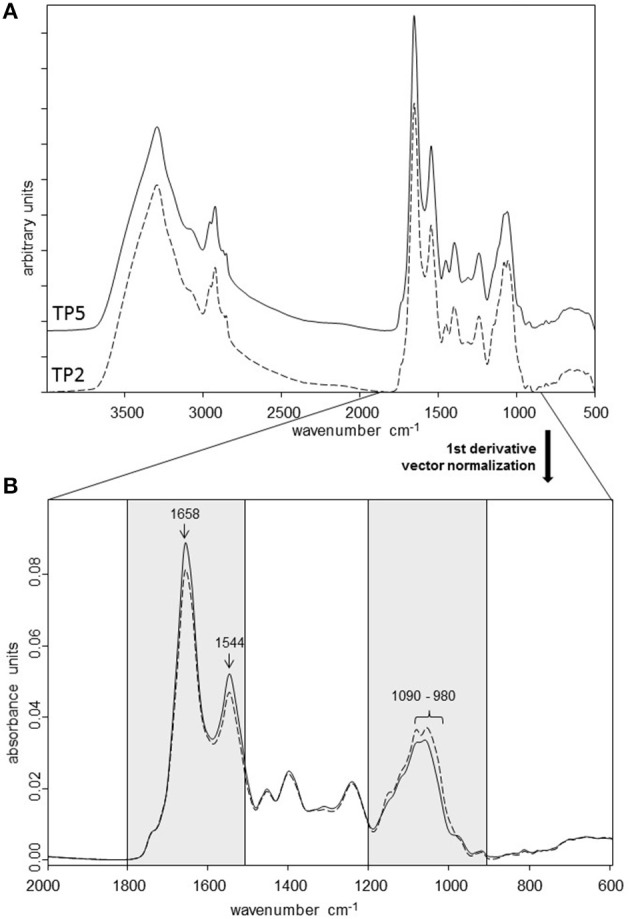
**(A)** FTIR spectra from TP2 and TP5 recorded in the transmission mode at 4,000 to 500 cm^−^^1^. First derivative, normalized average FTIR spectra of three independent measurements of TP2 and TP5. **(B)** Spectra are shown from the spectral range with the most pronounced differences between the two strains (amide/protein region: 1,800 to 1,500 cm^−1^; polysaccharide region: 1,200 to 900 cm^−1^).

FTIR spectroscopy was used as a high-resolution vibrational spectroscopic technique to generate metabolic fingerprints of the two *T. pyogenes* strains included in the study (Figure 2). FTIR spectral analysis of the average spectra generated from the multiple independent measurements of the strains revealed the most pronounced differences in the metabolic profiles of TP2 and TP5 in the amide/protein region (wavenumber 1,800 to 1,500 cm^−1^) and polysaccharide region (wavenumber 1,200 to 900 cm^−1^). The most prominent differences between the two strains were located at wavenumber 1,658 cm^−1^ (amide I region), 1,544 cm^−1^ (N = O bonds), and in the polysaccharide region at wavenumber 1,090 to 980 cm^−1^.

### Presence and mRNA expression of virulence genes

All the virulence genes investigated, except *nanP*, were present in the genome of the two *T. pyogenes* strains (data not shown). The *nanP* gene was only present in the bacterial genomic DNA of TP5, not of TP2.

According to the BLASTN analysis, the partial sequences of all virulence factors showed homologies to the sequences (GenBank accession numbers in parenthesis), as follows: *cbpA* 100% (AY223543.1), *fimA* 98% (CP007003.1 and CP007519.1), *fimC* 99% (CP007003.1 and CP007519.1), *fimE* 96–99% (CP012649.1, CP007003.1, and CP007519.1), *fimG* 98–99% (CP007003.1, CP007519.1, and CP012649.1), *nanH* 93–98% (AF298154.1, CP007003.1, and CP007519.1), *nanP* 97–100% (CP007003.1, CP012649.1, and AY045771.1), and PLO 97–99% (KJ150329.1, CP007519.1, and AB027461.1).

**Figure 3:**
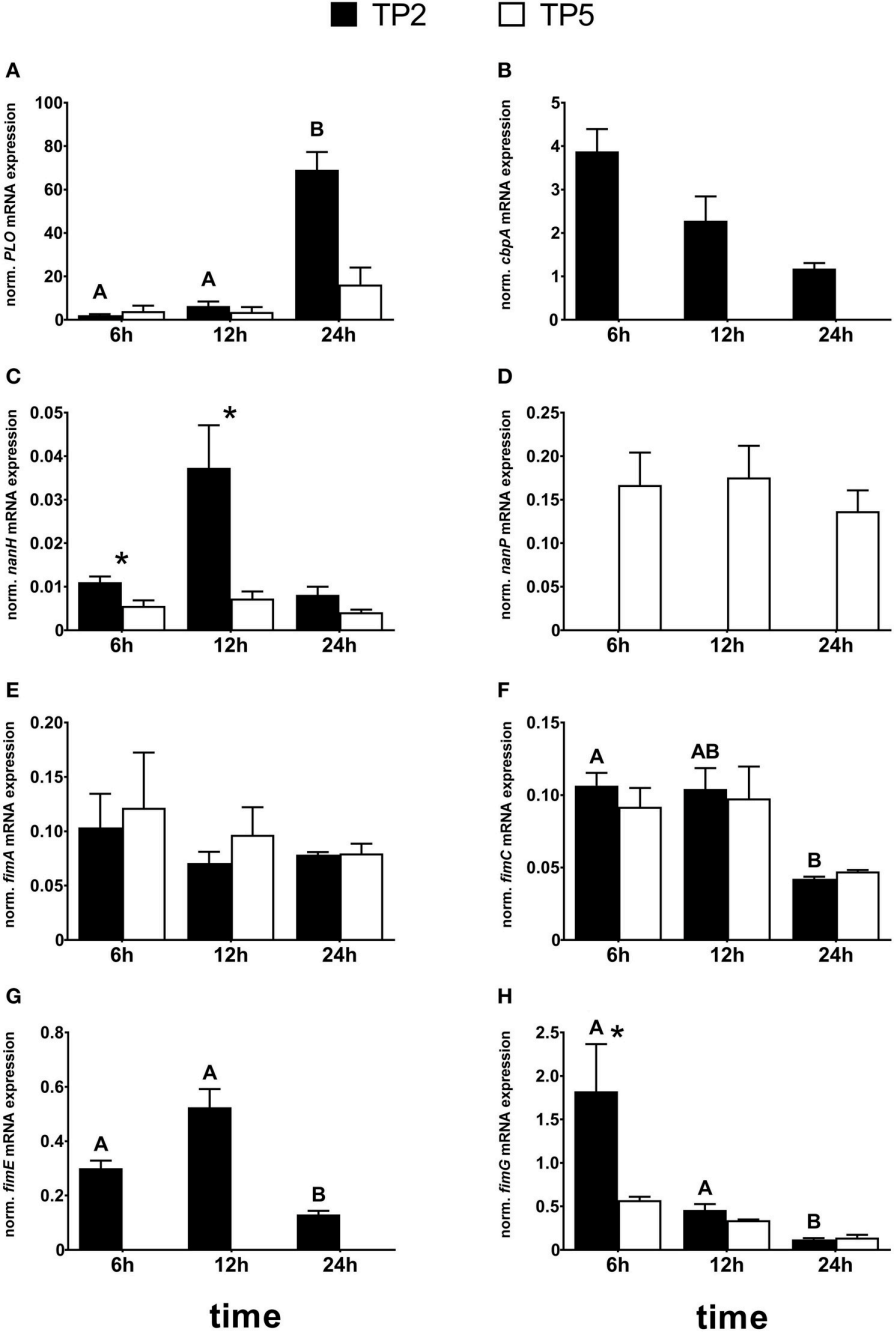
Normalized mRNA expression of the virulence factors: **(A)** *PLO*, **(B)** *cbpA*, **(C)** *nanH*, **(D)** *nanP*, **(E)** *fimA*, **(F)** *fimC*, **(G)** *fimE*, and **(H)** *fimG* in TP2 and TP5 cultured in BHI supplemented with 5% HI FCS up to 24 h. The bars show means ± SEM of mRNA expression values from different inocula (*n* = 3). Asterisks above the bars indicate significantly different mRNA expression (*P* < 0.05) between the two strains at the same time point. Different capital letters (A,B) above the bars indicate a significant difference within TP2 between different time points (*P* < 0.05).

Different mRNA expression of most virulence genes was noted between the two *T. pyogenes* strains (Figure 3). In detail, transcripts of *PLO* were detected during all time points with no significant differences between the two strains (Figure 3A). However, *PLO* mRNA expression was time-dependent in TP2 but not in TP5. mRNA expression of *PLO* in TP2 was significantly—20- and 7-fold—higher after 24 h compared with 6 and 12 h, respectively.

The *cbpA* gene was present in the genomes of both strains. However, *cbpA* mRNA expression was only detected in TP2, not in TP5 (Figure 3B), and showed a tendency for decreased mRNA expression after 24 h compared with 6 h (*P* = 0.057).

*nanH* mRNA expression was observed in TP2 and TP5 at all time points (Figure 3C). The transcription level of *nanH* in TP5 was similar during the 24 h cultivation. In contrast, *nanH* mRNA expression in TP2 increased 3-fold from 6 to 12 h, reaching a 4-fold lower level after 24 h. However, this finding failed to reach statistical significance (*P* > 0.1). After 6 and 12 h, *nanH* transcription in TP2 was significantly higher (*P* = 0.004 and 0.014, respectively) compared with TP5. *nanH* mRNA expression was 2-fold higher in TP2 than in TP5 after 24 h, but this difference did not reach statistical significance (*P* = 0.068).

Nearly, the same transcript amounts of *nanP* were detected in TP5 at the selected time points, but none in TP2 not containing this gene in its bacterial genome (Figure 3D).

Transcripts of *fimA* were detected in TP2 and TP5 at all time points investigated, with no significant differences between the two strains (Figure 3E). mRNA expression of *fimC* was observed during the entire cultivation time in both the *T. pyogenes* strains investigated (Figure 3F). A time-dependent *fimC* mRNA expression was noted for TP2, but not for TP5. The amount of *fimC* mRNA in TP2 was lower after 24 h (4-fold) compared with 6 h (*P* = 0.027) and 12 h (*P* = 0.057). Nearly the same *fimC* transcript amounts were detected in TP2 and TP5 after 6 and 12 h. However, mRNA expression of *fimC* after 24 h tended to be about 3-fold higher in TP5 compared with TP2, but failed to reach statistical significance (*P* = 0.051).

Similar to *cbpA*, the gene of *fimE* was present in the genomes of both strains. However, *fimE* transcripts were only detected in TP2, not in TP5 (Figure 3G). In addition, *fimE* mRNA expression was time-dependent and tended to increase (*P* = 0.06) from 6 h to 2-fold higher values at 12 h and decreased about 4-fold (*P* = 0.002) after 24 h compared with 12 h.

Transcripts of *fimG* were detected in TP2 and TP5 (Figure 3H). After 6 h, *fimG* mRNA expression was significantly higher in TP2 compared with TP5. No differential *fimG* mRNA expression was noted between the two strains after 12 and 24 h. The mRNA expression pattern of *fimG* in TP2 decreased with cultivation time, with about 15- and 4-fold lower *fimG* mRNA expression after 24 h compared with 6 and 12 h, respectively.

### Viability assay

**Figure 4:**
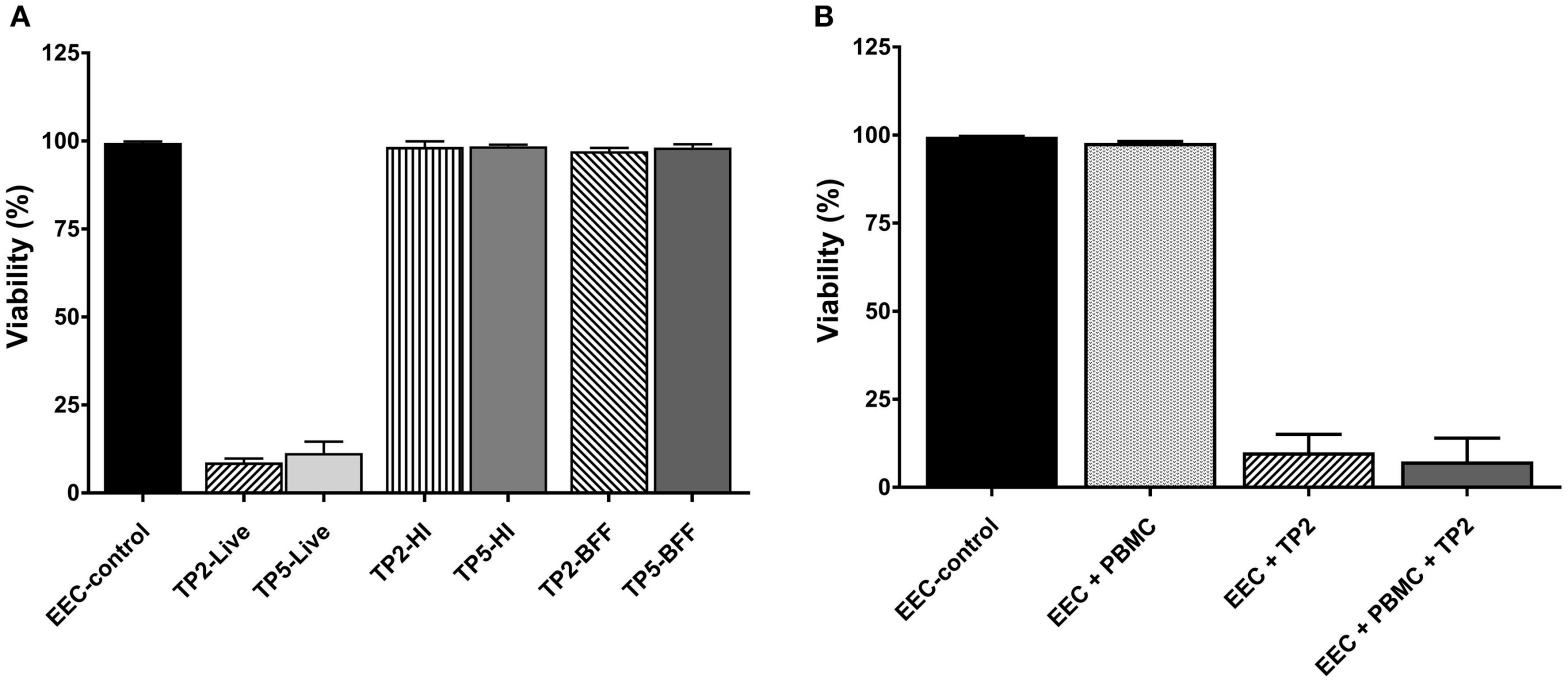
Percentage of viability of endometrial epithelial cells (EEC–control) **(A)** co-cultured with two strains of *T. pyogenes* (TP2 or TP5) in the form of live bacteria at MOI = 1 (−Live), or with heat-inactivated bacteria at MOI equivalent to 1 (−HI), or with bacteria-free filtrate at MOI equivalent to 1 (−BFF), and **(B)** co-cultured with live TP2 at MOI = 1 in the presence or absence of PBMC at a ratio of 1:1.

The effect of live bacteria, BFF (representing exotoxins), and HI bacteria (representing endotoxins) of both *T. pyogenes* strains on bovine endometrial epithelial cells was evaluated (Figure 4A). Similar cytotoxicity on endometrial epithelial cells was elicited by the live form of each of the two strains of *T. pyogenes*, which caused death in >90% of the cells within 16 h. This was not observed after 8 h, when >95% cells were still viable in treated and control epithelial cells (data not shown). In contrast, HI bacteria and BFF of the two strains did not elicit a cytotoxic effect on endometrial epithelial cells up to 72 h.

The viability of bovine endometrial epithelial cells co-cultured with the *T. pyogenes* strain TP2 isolated from a cow developing CE in the presence/absence of PBMCs was investigated (Figure 4B). More than 90% of the endometrial epithelial cells were dead after 16 h of co-culture with live TP2 alone, or with TP2 and PBMCs together, compared with the control. The presence of PBMCs did not affect the viability of endometrial epithelial cells compared with the control.

In addition, the viability of bovine PBMCs co-cultured with *T. pyogenes* strain TP2 at different MOI (0.1 and 1, respectively) was evaluated. After 8 h, the percentage of live PBMCs co-cultured with TP2 recovered at different MOI was lower compared with the percentage of recovered PBMCs incubated with control culture medium (data not shown). After 16 h, 100% of PBMCs were dead in the presence of TP2 at an MOI of 0.1 and 1 compared with control cells (data not shown).

### mRNA expression of pro-inflammatory factors in bovine endometrial epithelial cells co-cultured with two different *T. pyogenes* strains

**Figure 5:**
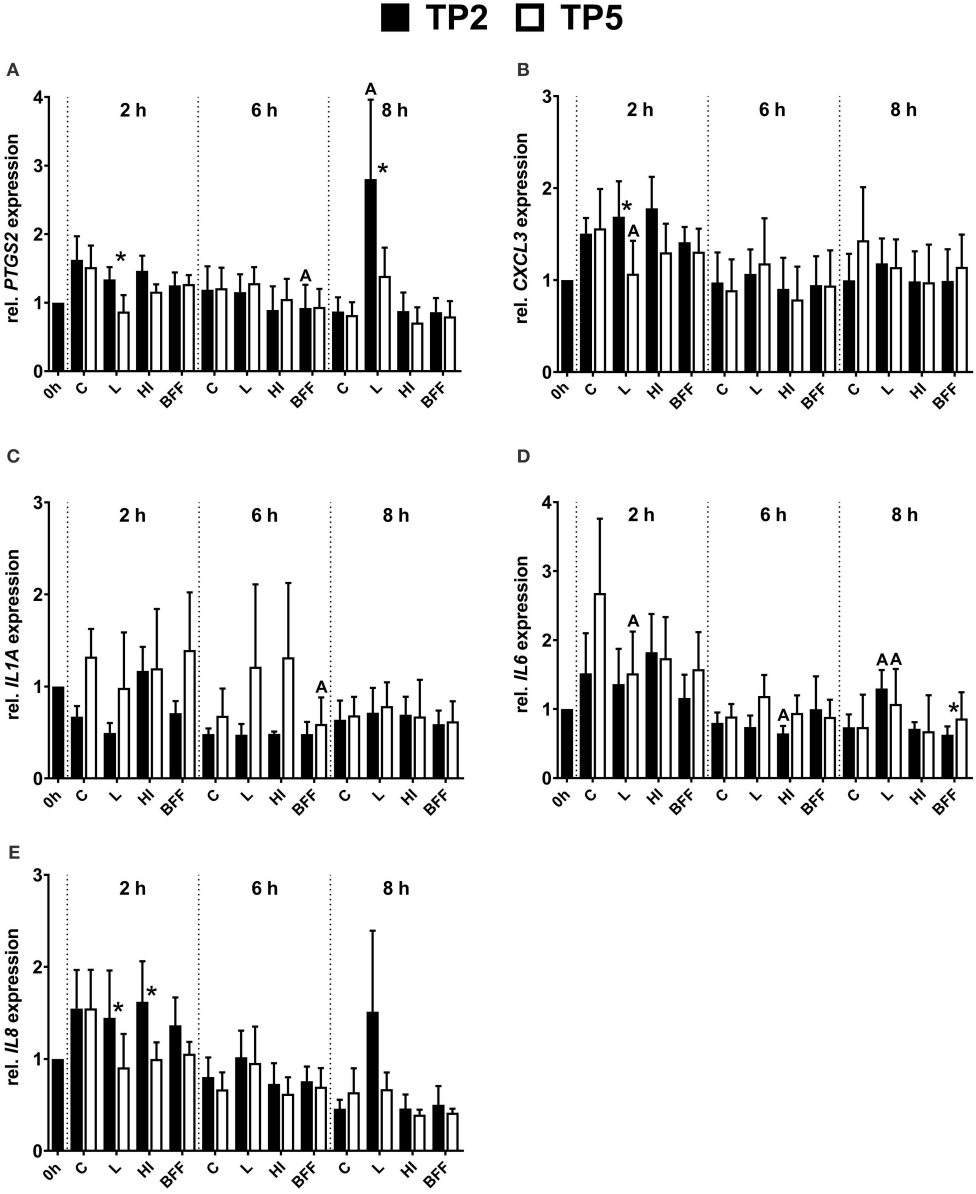
Relative mRNA expression of **(A)** *PTGS2*, **(B)** *CXCL3*, **(C)** *IL1A*, **(D)** *IL6*, and **(E)** *IL8* in bovine endometrial epithelial cells co-cultured with TP2 or TP5 at MOI = 1 up to 8 h in the form of live (L), heat-inactivated (HI), or bacteria-free filtrate (BFF). The bars show means ± SEM of mRNA expression values from different cultures (*n* = 5 cows). The letters above the bars indicate significantly different mRNA expression (*P* < 0.05) compared with the control (C; untreated) at the same time point. Asterisks above the bars indicate significantly different mRNA expression (*P* < 0.05) between TP2 and TP5 at the same time point.

The mRNA expression of *PTGS2* in endometrial epithelial cells was influenced significantly by the presence of TP2 but not by TP5 (Figure 5A). In detail, a significantly (3-fold) higher amount of *PTGS2* mRNA was observed in epithelial cells co-cultured with live TP2 after 8 h compared with the control. The *PTGS2* mRNA expression tended to decrease in the presence of live TP5 after 2 h (*P* = 0.08). In addition, the presence of live TP2 resulted in significantly higher *PTGS2* mRNA expression of endometrial epithelial cells, 1.5- and 2-fold after 2 and 8 h respectively, compared with live TP5. Endometrial epithelial cells co-cultured with TP2 BFF after 6 h showed a lower (*P* < 0.05) *PTGS2* mRNA expression compared with untreated cells. Furthermore, transcript amounts of *PTGS2* tended to decrease (*P* = 0.08) in the presence of HI or BFF TP5 after 6 h.

The presence of live TP2 did not affect *CXCL3* mRNA expression, with similar transcript amounts in endometrial epithelial cells compared with non-treated controls during the 8 h time period (Figure 5B). However, in presence of live TP5, significantly lower *CXCL3* mRNA expression was observed compared with controls after 2 h. Therefore, the mRNA expression of *CXCL3* in endometrial epithelial cells 2 h co-cultured with live TP2 was higher (*P* < 0.05) compared with live TP5.

During the 8 h co-culturing period, *IL1A* mRNA expression in endometrial epithelial cells was not significantly affected by the presence of live or HI TP2 and TP5, respectively (Figure 5C). However, lower *IL1A* mRNA expression (*P* < 0.05) was observed after 6 h in the presence of BFF TP5 compared with control cells, but not for BFF TP2.

*IL6* showed differences in the mRNA expression pattern in endometrial epithelial cells co-cultured with TP2 or TP5 at different time points (Figure 5D). After 2 h, *IL6* mRNA expression was significantly lower in the presence of live TP5 in comparison with untreated cells, but this was not observed for TP2. Significantly higher mRNA expression of *IL6* was detected after 8 h in the presence of live TP2 and live TP5 compared with control cells. Endometrial epithelial cells co-cultured with HI TP2 showed slightly lower (*P* < 0.05) *IL6* mRNA expression after 6 h compared with untreated cells. In contrast to the other pro-inflammatory factors, a lower mRNA expression of *IL6* in endometrial epithelial cells was observed in the presence of BFF TP2 compared with BFF TP5 after 8 h.

*IL8* mRNA expression in endometrial epithelial cells was similar in the presence of TP2 in the form of HI or BFF compared with control cells at each time point during the 8 h co-culture period (Figure 5E). However, *IL8* mRNA expression after 8 h tended to be higher (*P* = 0.08) in epithelial cells in the presence of live TP2 compared with the control. In addition, only a numerical decrease (*P* = 0.08) of *IL8* mRNA expression was observed after 2 h in presence of live TP5 in comparison with untreated cells. Endometrial epithelial cells showed a significantly higher *IL8* mRNA expression in presence of live and HI TP2 compared with live and HI TP5 after 2 h, respectively.

### mRNA expression of pro-inflammatory factors in bovine endometrial epithelial cells co-cultured with TP2 in the presence/absence of PBMCs

To monitor the absence/influence of any contamination with immune cells, mRNA expression of *CD45* was evaluated. Transcripts of *CD45* were not detected in almost all endometrial epithelial cell samples co-cultured with PBMCs. The melting curve analysis showed no or non-specific amplification except for six samples co-cultured with PBMCs, the threshold cycle value of which was >38 (data not shown).

**Figure 6:**
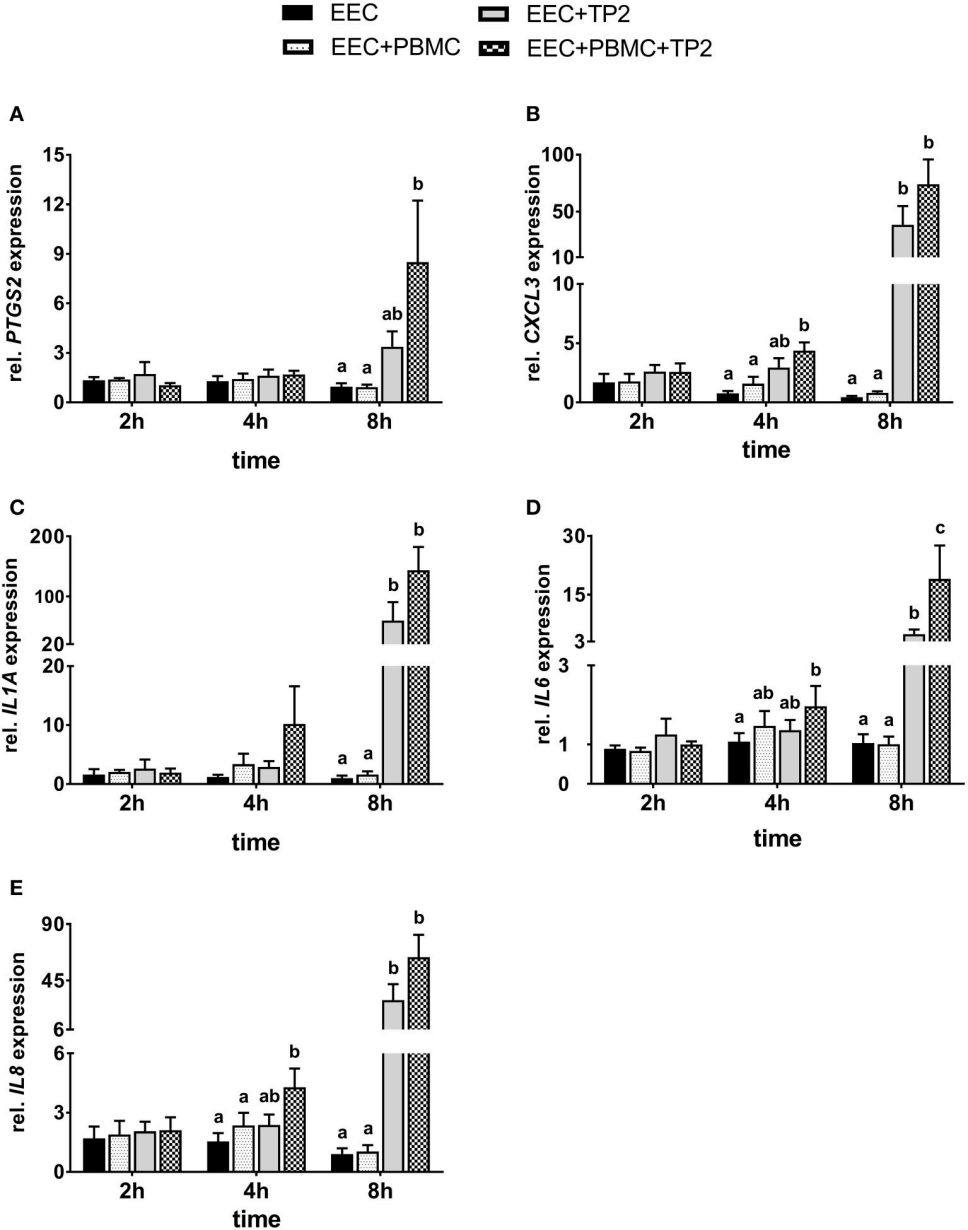
Relative mRNA expression of **(A)** *PTGS2*, **(B)** *CXCL3*, **(C)** *IL1A*, **(D)** *IL6*, and **(E)** *IL8* in bovine endometrial epithelial cells co-cultured with PBMC at a ratio of 1:1 and/or live TP2 at MOI = 1 up to 8 h. The bars show means ± SEM of mRNA expression values from different cultures (*n* = 5 cows). Different small letters (a,b,c) above the bars indicate significantly different mRNA expression between different treatments (*P* < 0.05).

Nearly, the same transcript amounts of *PTGS2* were noted in endometrial epithelial cells co-cultured with PBMCs compared with controls during different time points (Figure 6A). Endometrial epithelial *PTGS2* mRNA expression was 4-fold higher in presence of TP2 alone after 8 h compared with untreated cells controls but failed to reach a significant difference (*P* = 0.096). However, endometrial epithelial cells in the presence of TP2 and PBMCs showed significantly higher *PTGS2* mRNA expression (9-fold) after 8 h compared with controls.

The mRNA expression of *CXCL3* in endometrial epithelial cells was not affected significantly by the presence of PBMCs alone compared with untreated cells throughout the entire time course of co-culturing (Figure 6B). There was significantly higher *CXCL3* mRNA expression in endometrial epithelial cells co-cultured with TP2 and PBMCs compared with the untreated controls (*P* < 0.001), but not with TP2 alone (*P* = 0.17), after 4 h. However, greatly higher mRNA expression of *CXCL3* (50- and 90-fold) was observed in endometrial epithelial cells after 8 h co-cultured with TP2 compared with untreated cells and cells co-cultured with PBMCs, respectively. This effect was further amplified in endometrial epithelial cells in the presence of TP2 and PBMCs, which showed a 90- to 170-fold increase of *CXCL3* mRNA expression in comparison with controls.

There was no significant effect in the presence of TP2 or PBMCs, either alone or combined, on *IL1A* mRNA expression in endometrial epithelial cells after 2 h of co-culture (Figure 6C). After 4 h, epithelial cells co-cultured with TP2 and PBMCs showed an 8-fold higher *IL1A* mRNA expression compared with untreated cells. This increase failed to reach statistical significance (*P* = 0.06) due to the high variability between the animals. However, mRNA expression of *IL1A* in endometrial epithelial cells after 8 h was significantly higher with TP2 alone (35- and 60-fold), or TP2 and PBMCs (90- and 140-fold), when compared with untreated cells and cells co-cultured with PBMCs, respectively.

The *IL6* mRNA expression pattern was similar in treated endometrial epithelial cells and the controls after 2 h (Figure 6D). Although the mRNA expression of *IL6* did not increase in response to TP2 alone after 4 h, higher mRNA expression in epithelial cells (*P* < 0.05) was observed in the presence of TP2 and PBMCs compared with untreated cells. After 8 h, TP2 alone, or TP2 and PBMCs together induced higher *IL6* mRNA expression in endometrial epithelial cells compared with the controls. In addition, *IL6* mRNA expression in endometrial epithelial cells co-cultured with TP2 and PBMCs was 4-fold higher (*P* < 0.05) in comparison with epithelial cells co-cultured with TP2 alone after 8 h.

*IL8* mRNA expression exhibited no differences between treated endometrial epithelial cells and controls after 2 h (Figure 6E). In comparison with the control cells, higher *IL8* mRNA expression was observed in epithelial cells co-cultured with TP2 and PBMCs after 4 h. After 8 h, endometrial epithelial cells co-cultured with TP2 showed a 30-fold increase in *IL8* mRNA expression (*P* < 0.001); this increase was further amplified to 70-fold in the presence of PBMCs.

### mRNA expression of pro-inflammatory factors in PBMCs co-cultured with TP2 at lower concentrations

**Figure 7:**
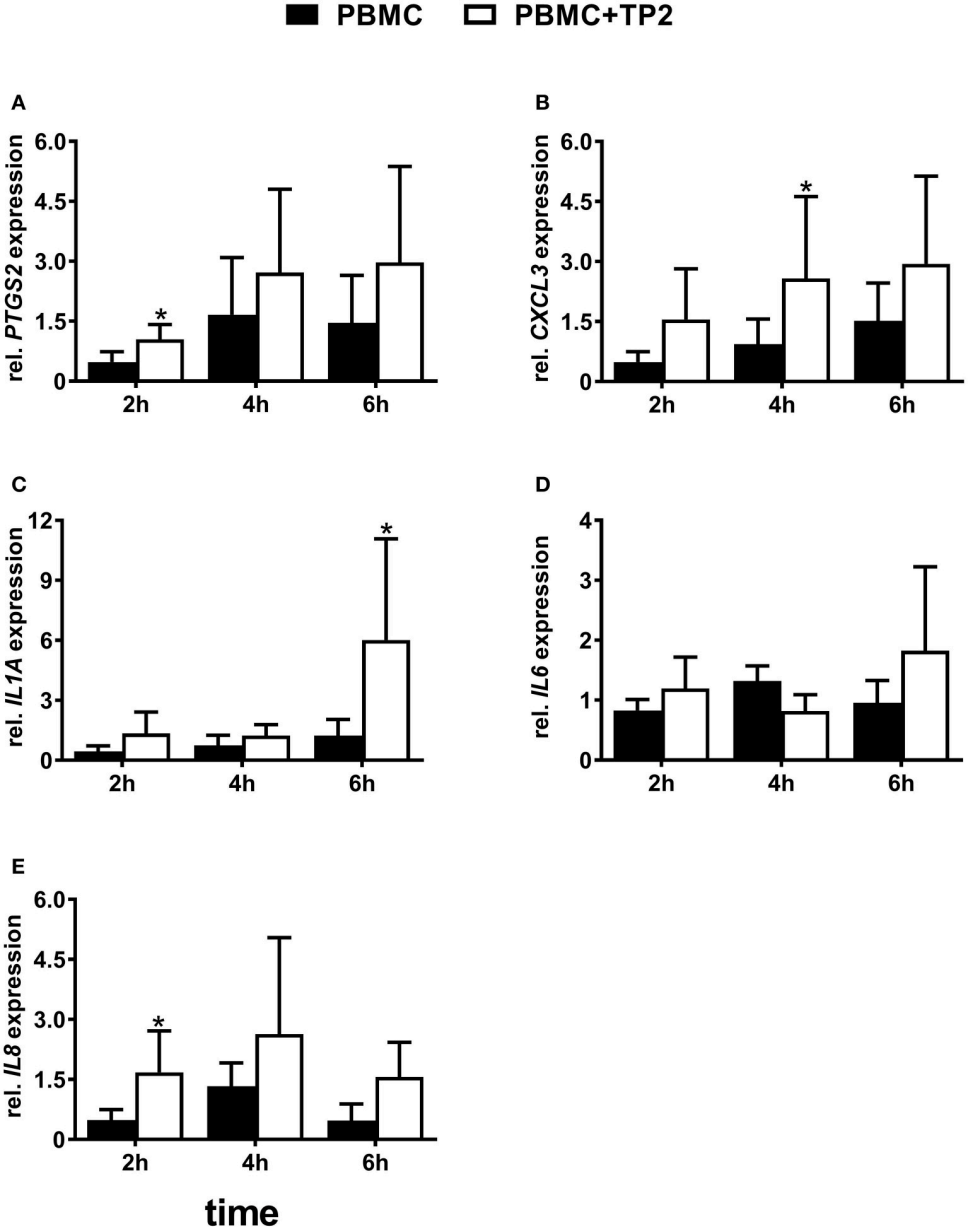
Relative mRNA expression of **(A)** *PTGS2*, **(B)** *CXCL3*, **(C)** *IL1A*, **(D)** *IL6*, and **(E)** *IL8* in PBMC co-cultured with live TP2 at MOI = 0.1 up to 6 h. The bars show means ± SEM of mRNA expression values from different cultures (*n* = 3 cows). Asterisks above the bars indicate significant different mRNA expression (*P* < 0.05) compared with the control (untreated) at the same time point.

PBMCs co-cultured with the *T. pyogenes* strain TP2 at MOI = 0.1 showed higher mRNA expression of some selected pro-inflammatory factors. In detail, PBMCs co-cultured with TP2 showed a 2-fold increase (*P* < 0.05) of *PTGS2* mRNA expression as early as after 2 h in comparison with the controls (Figure 7A), but not after 4 or 6 h.

The presence of TP2 affected the mRNA expression of *CXCL3* in PBMCs (Figure 7B). *CXCL3* mRNA was three times more highly expressed (*P* < 0.05) in PBMCs co-cultured with TP2 in comparison with the controls after 4 h. However, this increase was not observed at 2 or 6 h.

Nearly, the same transcript amount of *IL1A* was detected in PBMCs co-cultured with TP2 and in the controls after 2 and 4 h (Figure 7C). However, a significantly (5-fold) higher *IL1A* mRNA expression was observed in PBMCs co-cultured with TP2 in comparison with untreated cells after 6 h.

mRNA expression of *IL6* did not change significantly in PBMCs co-cultured with TP2 in comparison with controls at all investigated time points (Figure 7D).

Similar to *PTGS2*, higher *IL8* mRNA expression (*P* < 0.05) was noted in PBMCs treated with TP2 in comparison with controls as early as after 2 h (Figure 7E). Such higher mRNA expression was not observed at later time points.

## Discussion

This study followed the hypothesis that the occurrence of uterine diseases depends on bacterial virulence, as well as on the host immunity. Therefore, to assess the importance of strain-specific characteristics for *T. pyogenes* in bovine CE, two different *T. pyogenes* strains were included. One strain was isolated from the uterus of a cow developing CE (TP2) and the other strain was isolated from a healthy uterus (TP5). These strains differed in their growth characteristics, metabolic profiles, and mRNA expression pattern of virulence genes. Such findings might also explain similar observations that *T. pyogenes* strains have been found to be associated with severe uterine pathology (Sheldon et al., 2006; Lima et al., 2015), but have also been isolated from healthy uteri (Santos et al., 2010; Bicalho et al., 2012; Wagener et al., 2015).

The different initial immune reaction of bovine endometrial epithelial cells can be attributed to the differences in the metabolic profiles of the bacterial strains as revealed by FTIR spectroscopy. Notably, the most prominent differences were detected in the polysaccharide composition of both strains. Polysaccharides are an important component of bacterial peptidoglycans (PGN) and can initiate an innate immune response in host epithelial cells through toll-like receptors (TLR). It has been reported previously that bovine endometrial epithelial cells express TLR 1 to 7 and 9 (Davies et al., 2008). In addition, bovine endometrial epithelial and stromal cells can detect and respond to bacterial lipopetides found in gram-positive bacteria through TLR1, TLR2, and TLR6 (Sheldon et al., 2014; Turner et al., 2014). However, differences in such PGN patterns may lead to different recognition by TLR2 and subsequently different immune responses. This supports our observation that endometrial epithelial cells showed a higher pro-inflammatory response in the presence of TP2, which resulted after 8 h in increased *PTGS2* and *IL8* mRNA expression.

Furthermore, FTIR spectroscopy revealed differences in the protein region, which may be reflected in the different mRNA expression pattern of certain virulence factors between the two strains. The virulence factors cbpA, nanH, fimE, and fimG, which mRNA was more highly or exclusively expressed in TP2 compared with TP5, are involved in adhesion to host cells (Jost et al., 2002; Esmay et al., 2003; Jost and Billington, 2005). Therefore, TP2 might be able to attach better to epithelial cells by expressing more cell wall-associated virulence factors. Indeed, some *T. pyogenes* can invade the host cells, but usually *T. pyogenes* lives extracellular due to its ability to adhere to the host epithelium (Jost and Billington, 2005). This may reflect the importance of these virulence genes in TP2 pathogenicity.

These observations are consistent with antibodies detected against certain fimbrial proteins of *T. pyogenes* in cows with uterine *T. pyogenes* infection, reflecting the importance of fimbrial proteins for the development of postpartum uterine diseases (Bisinotto et al., 2016). *T. pyogenes* strains, which harbor a gene that encodes for *fimA*, have been found to be associated with metritis (Santos et al., 2010) and clinical endometritis (Bicalho et al., 2012). However, no relationship was found in another study between the presence of eight virulence genes of *T. pyogenes* and the development of clinical metritis (Silva et al., 2008). The ability of *T. pyogenes* to induce endometritis might be related to differential mRNA expression of their virulence genes, rather than simple presence in the bacterial genome. In the same context, most of the selected virulence factors in this study were expressed in a time-dependent manner in TP2.

PLO is the main virulence factor of *T. pyogenes* (Jost et al., 1999), but it may not be the only determinant factor for the pathogenicity of *T. pyogenes*. Both strains investigated caused the death of >90% of endometrial epithelial cells within 16 h of co-culture as living bacteria. This indicates that both strains are able to produce PLO, which causes cytolysis of endometrial cells. This goes together with our observation that *PLO* mRNA was expressed in both strains at different time points of bacterial growth without any significant differences.

Interestingly, the BFF and HI of the two strains did not influence the viability of endometrial epithelial cells up to 72 h, or the pro-inflammatory response. This may be due to the presence of a lower and sublytic concentration of PLO in the BFF compared with that produced by live bacteria. However, the BFF of 12 different *T. pyogenes* isolates caused cytolysis of endometrial cells in a previous study (Amos et al., 2014), which used a higher concentration of *T. pyogenes* (MOI = 1 vs. MOI > 10). The results of this study are also in line with previous findings showing that PLO in BFF or recombinant PLO did not stimulate a pro-inflammatory response in endometrial cells (Amos et al., 2014). However, endometrial cells generated a pro-inflammatory response to heat-killed *T. pyogenes* (Borges et al., 2012; Amos et al., 2014). This may be caused by the greatly higher number of bacteria used in these studies (equivalent to MOI = 1,000) compared with this study (equivalent to MOI = 1). This study intended to use similar starting material for a better comparison and also to reflect the situation at the beginning of a bacterial infection.

Furthermore, we investigated if culturing of endometrial epithelial cells with distinct peripheral immune cells influences epithelial cellular responses to *T. pyogenes* to gain insights into the role of host immunity for *T. pyogenes*-related uterine infections. Our study revealed that PBMCs alone did not influence the viability or pro-inflammatory response of epithelial cells. However, the presence of PBMCs amplified the pro-inflammatory response in bovine endometrial epithelial cells to pathogenic *T. pyogenes*. This pro-inflammatory response originates from epithelial cells rather than from any traces of other immune cells, the presence of which was monitored by *CD45* mRNA expression.

An important characteristic of the innate immune response is the fast reaction to a bacterial infection. However, the endometrial epithelial cells alone did not show a reaction to live *T. pyogenes* strain TP2 up to 6 h of co-culturing in this study compared with an early reaction after 2 h to *Bacillus pumilus* (Gärtner et al., 2016). In contrast, endometrial epithelial cells in the presence of PBMCs responded to *T. pyogenes* strain TP2 as early as after 4 h by increasing the mRNA expression of *PTGS2, CXCL3, IL6*, and *IL8*. Furthermore, immune cells have low thresholds in the response to bacteria and their patterns (Shaykhiev and Bals, 2007). In this study, PBMCs reacted to *T. pyogenes* at a level as low as MOI = 0.1 through the increased mRNA expression of *PTGS2* and *IL8* after 2 h. These data suggest a mechanism whereby leukocytes within the endometrium may sensitize the epithelial cells to initial bacterial invasion by up-regulating the uterine innate immune response. As PBMCs alone did not influence the epithelial cells, we hypothesize that the effect of immune cells on epithelial cells may be mediated by the release of soluble factors from bacteria-activated immune cells rather than by cell-cell contact (Panja et al., 1994). Interestingly, *IL6* mRNA expression in endometrial epithelial cells was 4-fold higher in the presence of *T. pyogenes* strain TP2 and PBMCs compared with TP2 alone after 8 h. This higher induced expression may be mediated by the release of soluble factors, such as *IL1A* from *T. pyogenes*-activated PBMCs. This is supported by our findings that PBMCs co-cultured with *T. pyogenes* strain TP2 showed higher *IL1A* mRNA expression. *IL1A* released from damaged endometrial cells bound to the *IL1* receptor 1 on nearby endometrial cells, stimulating further secretion of *IL6* (Healy et al., 2014). However, this study assumes that *IL1A* derived from bacteria-activated immune cells rather than from damaged endometrial cells leads to up-regulation of a uterine inflammation. Indeed, measuring protein level of IL1A in culture supernatants will be interesting to follow the signaling cascade within the endometrium to attract immune cells. However, the limitation of the current set up was that the bacteria killed the epithelial cells within 16 h.

Uterine infections are associated with disturbed corpus luteum (CL) function due to the disturbance of PGE_2_:PGF_2α_ ratios (Miller et al., 2007; Herath et al., 2009). Intrauterine infusion of live *T. pyogenes* has been shown to diminish the life span of the CL (Kaneko et al., 2013; Lima et al., 2015) or prolong its life span (Farin et al., 1989). This study indicates that PBMCs might up-regulate the mRNA expression of *PGTS2*, which is a key enzyme of PG synthesis, in endometrial epithelial cells in response to *T. pyogenes*. This up-regulation might be mediated by *IL1A* because *T. pyogenes* also induce higher *IL1A* mRNA expression in PBMCs. This is in line with earlier findings that PGE_2_ and PGF_2α_ synthesis in bovine endometrium throughout the estrous cycle is modulated by *IL1A* (Tanikawa et al., 2005). These findings suggest that *T. pyogenes* may disturb the CL function through up-regulation of a key enzyme involved in PG synthesis.

## Conclusion

This study supports the hypothesis that the pathogenicity of *T. pyogenes* can be attributed to certain strain characteristics. Notably, the clinical *T. pyogenes* strain included in this study shows not only a higher expression of known virulence factors compared with a *T. pyogenes* strain isolated from a healthy cow, but also distinct differences in the metabolic profile and growth characteristics. Therefore, certain strains of *T. pyogenes* could be an important factor for the development of endometritis in dairy cows after parturition. In addition, the presence of immune cells amplifies the pro-inflammatory response in endometrial epithelial cells to pathogenic *T. pyogenes*. All mRNA expression data in this *in vitro* model reflect the *in vivo* situation by up-regulation of such pro-inflammatory factors in cases of subclinical/clinical endometritis (Gabler et al., 2009; Fischer et al., 2010; Peter et al., 2015). However, further studies are needed to elucidate the detailed mechanisms and factors involved in communication between immune cells and endometrial cells during an infection; also, the exact role of cell wall-related factors in the host–pathogen interplay need to be deciphered.

## Author contributions

MI: Contributed ideas, performed experiments, analyzed data, and wrote the manuscript; SP: Performed experiments and edited the manuscript; KW: Performed experiments, analyzed data, and wrote the manuscript; MD: Contributed ideas, contributed to the clinical studies, provided bacterial strains, and edited the manuscript; RE: Contributed ideas and edited the manuscript; ME-S: Contributed ideas, undertook bacterial strain analysis, and edited the manuscript; CG: Conceived the study, designed experiments, analyzed data, and wrote the manuscript.

### Conflict of interest statement

The authors declare that the research was conducted in the absence of any commercial or financial relationships that could be construed as a potential conflict of interest.
